# Middle-School Student Engagement in a Tick Testing Community Science Project

**DOI:** 10.3390/insects12121136

**Published:** 2021-12-18

**Authors:** Amy Prunuske, Cole Fisher, Jhomary Molden, Amarpreet Brar, Ryan Ragland, Jesse vanWestrienen

**Affiliations:** 1 Department of Microbiology and Immunology, Medical College of Wisconsin-Central Wisconsin, Wausau, WI 54401, USA; 2 Department of Biomedical Sciences, University of Minnesota Duluth, Duluth, MN 55812, USA; nfisher@d.umn.edu; 3 Department of Biology, University of Wisconsin Stevens Point, Stevens Point, WI 54481, USA; jhomary22@gmail.com (J.M.); amarbrar11@gmail.com (A.B.); 4 Biomeme, Philadelphia, PA 19107, USA; ryan.lee.ragland@gmail.com (R.R.); jesse@biomeme.com (J.v.)

**Keywords:** citizen science, Lyme disease, *Ixodes scapularis*, tick, *Borrelia burgdorferi*, community science, *Anaplasma phagocytophilium*, *Babesia microti*

## Abstract

**Simple Summary:**

Lyme disease is a common tickborne illness endemic to many countries, including the United States. Scientists have a role to play in disseminating public health knowledge to decrease the prevalence of tickborne disease, which can include encouraging preventive behaviors and recognizing the early signs of the disease. Middle-school students are at significant risk of developing Lyme disease and an ideal population to engage in community-based science, since these experiences provide valuable opportunities for career explorations and to extend the students’ understanding of science. Our work shows that the students can meaningfully contribute to research by generating samples that can be used to test whether the ticks contain pathogens.

**Abstract:**

Studies of tickborne illness have benefited from interactions between scientists and community members. Most participants in community science projects are well-educated adults, but there are anticipated benefits from engaging younger students in research. We evaluated whether an outreach experience for rural middle-school students promoted student interest in science and resulted in the generation of samples that could be used for tick testing to assess disease risk. Middle-school students from 78 Wisconsin communities developed interdisciplinary hypotheses about the spread of Lyme disease, identified ticks, and extracted DNA from ticks to assess the prevalence of pathogens *Borrelia burgdorferi*, *Anaplasma phagocytophillium*, and *Babesia microti*. As a result of this intervention, students were able to successfully complete the research protocol and explain the rationale for completing the experiment. Of student participants, 84.7% reported no difficulty completing the protocol, 66% of the student samples gave reliable PCR results, and 76% of students reported interest in participating in similar experiments. Our study shows that tick outreach programs that incorporate community-based science promote knowledge about Lyme disease, facilitate engagement between students and scientists, and generate samples that can be successfully utilized for pathogen testing.

## 1. Introduction

In the 1970s, two mothers from Lyme, CT collected local data that they shared with scientists leading to the discovery of Lyme disease [1]. This demonstrated a role for citizen or community science, which has only grown in recent years and facilitates the generation of data by amateur collectors [2]. In Wisconsin, Lyme disease is transmitted through tick vector *Ixodes scapularis,* and there is growing concern about the prevalence of *I. scapularis* carrying not only Lyme disease pathogen *Borrelia burgdorferi*, but also pathogens *Babesia microti* and *Anaplasma phagocytophila* [3]. Assessing the risk of tickborne illness through environmental surveillance fits under the One Health model that requires a holistic understanding of how the environment, animal health, and human health form a connected public health web [4]. New opportunities for community science are facilitated by the invention of portable scientific tools coupled to mobile technology that support the testing of samples for pathogens outside of the laboratory. These technologies support wider and more inclusive participation in science, but raise questions about the reliability of community-generated data and analysis as compared to experiments completed by trained scientists.

Community engagement is strengthened when tied to local context, which is why we developed community science presentations focused on tickborne diseases targeted at rural middle-school students. Middle-school students were selected as the target population since they are a high-risk group for the development of tickborne illness [5]. Middle school is also a time when students are beginning to explore careers and could benefit from engagement in the practice of science, including developing hypotheses and completing experiments. These types of experiences help students in building their science identities or core sense of the value of science [6]. Many rural students have never met a scientist and are unfamiliar with the pathway to garnering an advanced degree in science [7]. A potential challenge in using middle-school students is the variability in their prior experience completing scientific protocols. 

Attending a Lyme disease outreach presentation had helped students in increasing their knowledge of Lyme disease and ability to recognize ticks that transmit Lyme disease [8]. We next wanted to determine whether students could successfully extract DNA from ticks to be used as part of a community science research project designed to characterize the prevalence of the pathogens in ticks from Wisconsin. This experiment was designed for optimal learning by considering the middle-school audience, adopting purposeful activity, incorporating social interaction, and optimizing activities on the basis of participant feedback [9]. To evaluate the effectiveness of the intervention, we characterized who participated in the project, viewed videos of students completing the DNA extraction protocol, assessed the student understanding of why the procedure was completed, and determined the percentage of samples that generated usable research results.

## 2. Materials and Methods

### 2.1. Evaluation of Tick Outreach Sessions

Presentations were primarily held at the University of Wisconsin–Stevens Point as part of Science Technology Engineering and Math and College for Kids outreach events held in 2017–2019, targeting middle-school students aged 11–14 years. Three to four sessions were delivered in a day for groups of 20–25 students. A faculty member and 1–2 undergraduates delivered the interactive session, giving middle-school students the opportunity to answer multiple-choice polling questions and to ask their own questions about Lyme disease. Participants were asked to hypothesize what might contribute to changing the epidemiology of tickborne illnesses in Wisconsin and to discuss how to test their hypotheses, including by testing the ticks for pathogens. Then, the middle-school students extracted DNA from ticks to generate samples, and the presenter emphasized that the undergraduates would analyze their samples with a portable RT-PCR device. After completing the activity, students completed a reflection worksheet (Supplemental Materials) which was reviewed and independently coded by the faculty member and an undergraduate to identify broad themes that were discussed and refined using a grounded theory approach.

Google Maps (https://www.google.com/maps/, accessed on 26 February 2019) was used to show the distribution of schools participating in the presentation. For a subset of the presentations, we collected consent forms from parents to review videos of the students completing the DNA extraction protocol. The faculty member and undergraduates collaborated to identify student errors that had been observed and behaviors that supported the successful completion of the DNA extraction. The protocol was approved by the Institutional Review Board of Medical College of Wisconsin (PRO00029271).

### 2.2. Tick Identification

**Figure 1:**
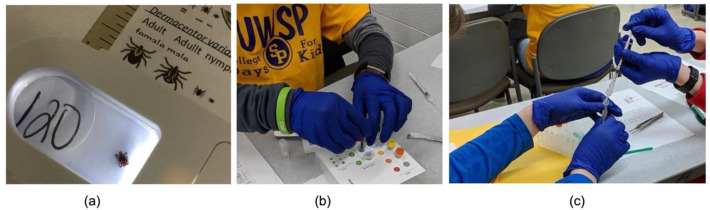
Students identifying tick species and performing DNA extraction. (**a**) Students identify an adult female *I. scapularis* using a magnifying glass. (**b**) Students use a pestle to crush tick in the lysis buffer. (**c**) Students use a syringe to purify the DNA.

Undergraduates assisted the middle-school students in identifying the ticks and served as role models by sharing with students their own path to their undergraduate degree [10]. In groups of 2–4, the middle-school students used a tick key (Minnesota Department of Health) to identify the species and sex of a tick. Community members removed the ticks from dogs through passive surveillance or, prior to the session, undergraduates engaged in active flagging using drag cloths, which led to the generation of a geographically and temporally diverse tick bank, and the researchers noted any relevant travel history. Ticks were stored in a 1.5 mL Eppendorf tube containing 750 µL of alcohol-based hand sanitizer (at least 60% w/v in water) at −20 °C so that they would not dry out prior to the middle-school students’ analysis. The faculty member generated an ID number, and recorded the species, sex, and location where the tick was collected prior to the session to ensure that we had accurate data for our records. Most of the ticks selected for testing were *I. scapularis,* the predominant vector for transmission, and a small subset of students viewed *Dermacentor virabilis,* the other tick species that is commonly found in Wisconsin. The students took pictures of their ticks and the corresponding ID number using iPads (Figure 1). Students utilized personal protection, including gloves and safety googles. The faculty member emphasized to the participants that the ticks were dead, and that transmission requires the ticks to be attached to the host for an extended period of time.

### 2.3. Extracting DNA from Ticks

The DNA of adult ticks and any pathogens it contained were extracted by the participants using a lysis protocol developed by Biomeme, Philadelphia, PA [11]. A written protocol was provided to the participants, and the procedure was completed by the faculty member or an undergraduate at the front of the classroom. A subset of students recorded their groups carrying out the protocol using the same iPad that they used to take pictures of the ticks. The first step in the DNA extraction protocol was to disrupt the tick cellular material by using a pestle in a lysis buffer, releasing the DNA from the cells (Figure 1). The crushed tick is allowed to settle to the bottom of the lysis buffer tube. The liquid containing the DNA was drawn up with a 1 mL syringe attached to a column containing a silica membrane that selectively binds the DNA, and pushed back out. This was repeated for 10 pumps. The column was washed to remove unwanted cellular material with a protein wash, wash buffer to remove salts, and drying buffer (1 pump each). Lastly, the column was air-dried (20 pumps). and the purified DNA was eluted off the membrane as part of the final step (5 pumps).

### 2.4. Real-Time PCR Assay

After the presentation in the laboratory, the undergraduates added 20 µL of the purified DNA to each of 3 wells containing lyophilized primers, 2 fluorescent probes (SYBR/Cy5), and a master mix. The assay allows for confirmation of the tick species by testing for *I. scapularis*, *D. variabilis*, and *A. americanum,* and to test for most prevalent tickborne pathogens *B. burgdorferi*, *A. phagocytophilum*, and *B. microti*. Species identification through PCR was used as a positive control, since the tick species for each sample was known. The test strip was placed in the portable Biomeme two3, a thermocycler attached to an iPhone [11]. Samples are initially denatured for 1 min at 95 °C, then denatured for 1 s at 95 °C, and annealed for 20 s at 60 °C for 45 cycles. The cycle threshold (Ct) was determined, and data were then uploaded to an online portal. Strategies to prevent DNA contamination of samples included using lyophilized reagents, decontaminating surfaces with 70% ethanol, using aerosol-resistant filtered pipette tips, not opening tubes containing the amplified product, and properly disposing of all tubes [12].

### 2.5. Real-time PCR Confirmation 

Data collected with the Biomeme device were verified using the Roche LightCycler^®^ 480 instrument (software version LCS480 1.5.0.39; Roche Diagnostics, Mannheim, Germany). RT-PCR reactions were conducted in LightCycler^®^ 480 Multiwell Plate 96, white plates (Roche Diagnostics, Mannheim, Germany). Total reaction volumes of 20 µL comprised up to 8 µL of DNA, either 10 µL Biomeme MasterMix or 1 × LightCycler^®^ 480 Probes Master (Roche Diagnostics, Mannheim, Germany), either 1.0 µL *A. phagocytophilum*-CY5 probe or *B. microti*-CY5 probe, 1.0 µL *B. burgdorferi*-FAM probe, and PCR-grade water. The LightCycler^®^ 480 instrument standard factory template for Dual Hydrolysis Probe-UPL Probe 96-II settings with FAM (465–510 nm) and CY5 (618–660 nm) were used to program assay parameters. Initial enzyme activation was set at 95 °C for 10 min, followed by 45 cycles of amplification run at 95 °C for 10 s, 60 °C for 30 s, and 72 °C for 10 s. The assay was completed with a cooling cycle at 40 °C for 30 s. Known samples of *B. burgdorferi, A. phagocytophilum,* and *B. microti* DNA were utilized as positive controls. Roche LightCycler^®^ 480 instrument software Abs Quant/2nd Derivative Max was used to calculate the Ct value.

## 3. Results

**Figure 2:**
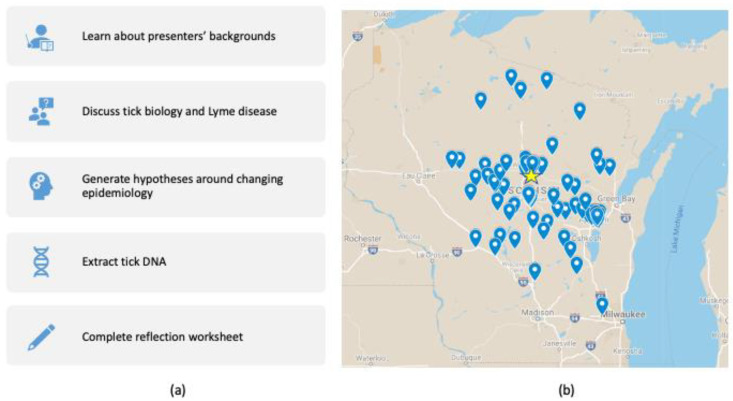
Middle-school community science session incorporated students from across the state. (**a**) Description of activities that were included in the session. (**b**) Map displaying the location of the schools that participated in the outreach activity. Star indicates the location of the university that hosted many of the presentations.

The objectives of the Lyme disease community science session were to expose middle-school students to science careers, to analyze information about tick biology and Lyme disease, and to generate DNA samples for tick testing (Figure 2a). Over 400 middle-school students (age 11–14 years) participated in the session with approximately equal participation by gender. By partnering with a local university, we were able to reach a wide geographic distribution of middle-school students from 78 different schools and all from small metropolitan areas of fewer than 150,000 residents (Figure 2b). This included students from groups under-represented in science, including the Menominee Indian Nation, Stockbridge-Munsee Community band, and Hmong refugees. Middle-school students then worked with the undergraduates to take pictures of the tick, to identify the tick species, and to extract DNA (Figure 1). Capturing high-quality images was one aspect that was optimized over the program, first utilizing magnifying glasses, and ultimately benefitting from improved cameras in the more recent iPad models. Students developed hypotheses about why the number of tickborne illnesses has been increasing in North–Central Wisconsin, and completed a reflective worksheet at the end of the session to check if students understanding the goal of the experiment (Supplemental Methods).

We reviewed 10 videos of student teams completing the DNA extraction protocol. It took the students 10–15 min to complete the DNA protocol as a group of 2–4 students. Students were given a written protocol, but very few students were observed looking at the written protocol, with most following along with the presenter at the front of the classroom. Students worked in groups, and often asked each other and the undergraduates for clarification: “So what do you do after?” and “Is there DNA in every single one of these (the tubes)?” Often, one student would open and close the tubes, while the other pumped or the participants took turns performing the steps, so there was active engagement of all participants. Students made relevant observations, including discussing the presence of a white precipitate on the pestle, the smell of the ethanol in one of the reagents, and noticing differences in the shape of the reagent tubes because of differences in the volume of liquid they contained. Students also corrected each other. In one case, a student was trying to use the pipette instead of the pestle to crush the tick: “This is actually what we are supposed to use.” Most of the students appeared to have completed each of the steps in the protocol and avoided contamination of the samples. 

Students were asked on the worksheet to indicate whether they had trouble completing the DNA extraction protocol, and 84.7% reported no difficulty completing the protocol (Table 1). Students indicated “Not really, you just needed to follow directions.” The students had the most challenges with crushing the tick due to the tough carapace, and we stopped using nymphs since they were harder for the students to transfer due to their small size. The greatest challenges were spilling the sample and using the buffers in the wrong order. Some students did not completely expel the liquid before moving on to the next tube and instead transferred liquid between tubes, potentially diluting the buffers and negatively impacting yield. The presenters began to emphasize the importance of fully expelling the sample before moving on to the next tube. It was also helpful to prelabel the tubes to ensure that the undergraduates could correctly match up the samples after the presentation.

When students were asked on the worksheet “Why did you complete the DNA extraction protocol?”, 58.6% understood that the goal was “to test whether the tick can transmit Lyme disease”; some went further: “wanted to figure out if ticks could kill us”. Of the students, 14.3% responded that they had completed the experiment “because you told us to”. Other answers included more aspirational ideas, because “it was fun and interesting”, “we wanted to advance science”, or because “I want to work in the medical field”. In addition, 76.1% indicated they would like to participate in another community science project while only 47.5% indicated they were interested in a career in science. “I’m not sure because I love doing different experiments, but I don’t know if I want that to be my job.” Students were unsure of how science might fit with their future career progression, and indicated not being interested in a science career even when there was a high likelihood that their career interest would require some science familiarity (i.e., being a doctor or fishing guide). The second two statements were added to the reflection worksheets midway through the presentations, which is why the number of responses were slightly lower.

After the presentation, the samples generated by the middle-school students were tested for *Borrelia burgdorferi*, *Anaplasma phagocytophilum*, and *Babesia microti* by the undergraduates using the portable RT-PCR device. The assay also confirmed the tick species differentiated among *Ixodes scapularis*, *Dermacentor varabilis*, and *Amblyomma americanum*. Of the 92 tested *Ixodes scapularis* samples, 61 samples (67%) successfully amplified *Ixodes scapularis* DNA. From the samples that were successfully amplified, 41% contained *Borrelia burgdorferi* DNA, 9.8% contained *Anaplasma phagocytophilum*, and 4.9% contained *Babesia microti* (Table 2). Results obtained with the portable RT-PCR device were confirmed with the Roche LightCycler, and these rates fell within the range of those that had been observed in previous studies in Wisconsin. 

## 4. Discussion

By partnering with a local college, over 400 rural middle-school students had the opportunity to participate in a community science project and generate samples that contributed to a larger scientific effort. Several components of the design facilitated active bidirectional knowledge exchange, with participants not just learning from scientists about how to prevent tickborne disease, but also offering up their own hypotheses and experiences with Lyme disease. One of the advantages of community science is questioning pernicious assumptions about who is capable of participating in science [9]. Engagement with students from rural backgrounds is important, since rural students are less likely to have access to camps or informal science learning environments such as museums and to enroll in postsecondary science, technology, engineering, and mathematics (STEM) degree programs, compared with their suburban peers [14]. Empowering wider participant engagement in community science has the potential to increase community science literacy or the ability to use science to advance local community issues [9].

Key partnerships facilitated the authentic participation of students in the work of scientists. Others have highlighted the value of partnerships among universities, students, and teachers to successfully doing community-based work [15], but our work shows that incorporating industry partners adds additional value that can offset resource and logistical costs. Our evaluation demonstrated that community members can effectively process samples for accurate pathogen testing, which is important to avoid misleading conclusion. Our methods facilitated the identification of some design flaws in the DNA extraction kit that contributed to loss of the sample, resulting in new kits coming in cartridge format. Given our results showed that the portable RT-PCR device generated similar results to those of laboratory-based DNA testing, there is increased opportunity to engage additional community members in this technology, which has also become more relevant with the increase in DNA-based testing during the COVID-19 pandemic [16]. The company is developing a shared platform to integrate the results from around the world, allowing for participants to look up their individual results paired with visual data on a map [17]. Over the long term, this could be used to crowdsource the monitoring of geographic and temporal changes in the pathogens, and allow for participants to perform their own data analysis, which would require additional training and longer-term engagement between the scientist and a subset of students. 

This study focused on middle-school students who volunteered to participate as part of a local college field trip. It is important to know your audience, and identify obstacles that might negatively impact motivation and limit the generalizability of the results. Others found that the majority of citizen science experiences for students do not meet next-generation standards with students struggling to understand *why* they are collecting data [18]. We aligned our activity with these standards, and the reflection worksheet allowed for students to articulate in their own words the purpose of the activity. Best practices for that age group included incorporating opportunities to utilize technology and interact with peers and near-peers (undergraduates). There may be tension between students having fun and ensuring that they generate samples of sufficient quality. Most of the samples resulted in amplification of the control target, but not all; so, we recommend that samples be processed by trained scientists if they are limited. Follow-up studies would support a better understanding of the long-term impact of the experience, and there could be more deliberate partnerships with teachers in the development of additional curricula. Others found that teachers that participate in insect outreach activities are subsequently much more interested in including insect-focused content [19].

Successful community engagement requires mutually beneficial partnerships, which was necessary for facilitating a large-scale community science endeavor [20]. Public engagement is encouraged for all federally funded research, and our work provides recommendations and strategies for scientists to utilize with middle-school students to advance scientific and participant goals.

## Figures and Tables

**Table 1:** Summary of reflection worksheet.

Statement	Yes	Maybe	No
1. Trouble completing the protocol *n* = 308	13.6%	1.6%	84.7%
2. Interest in participating in another community science project *n* = 117	76.1%	8.5%	15.3%
3. Interest in a career in science *n* = 267	47.5%	14.2%	38.3%

**Table 2:** Pathogen testing results. Percentage of adult ticks that tested positive for each of the pathogens from this study (Prunuske) and from similar studies conducted in Wisconsin (Stauffer and Westwood).

	Prunuske *N* = 61	Stauffer *N* = 36 [13]	Westwood *N* = 461 [3]
*Borrelia burgdorferi*	41.0%	52.8.%	17.4%
*Anaplasma phagocytophilum*	9.8%	5.6%	14.3%
*Babesia microtii*	4.9%	2.8%	6.5%

## Data Availability

The data presented in this study are available on request from the corresponding author.

## Supplementary Materials

The following are available online at https://www.mdpi.com/article/10.3390/insects12121136/s1, Figure S1: Handout used to assess student understanding of activity.
