# Supplementary material for: Middle-School Student Engagement in a Tick Testing Community Science Project
Source: Insects. 2021 Dec 18;12(12):1136. doi: 10.3390/insects12121136 (PMC8708189; doi:10.3390/insects12121136)
Supplement: Supplementary file 1 [file insects-12-01136-s001.zip › insects-1435646-supplementary.pdf]

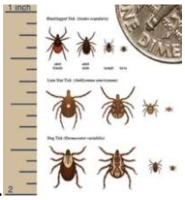

.

Remove tick using tweezers, place the tick below with the number of your sample, and take photo with iPad.

Circle the species of your tick: *Ixodes scapularis Dermacentor variabilis* Not sure

Circle the life stage/sex of your tick: adult male adult female nymph

Is it possible for you to get Lyme disease from this tick?
Circle your answer Yes No Not sure

If you want to be part of the study, set-up the iPad and record you and your partner(s) completing the DNA extraction protocol.

After completing the extraction, answer the following questions.

Did you have any trouble completing the protocol?

Why did you complete the DNA extraction protocol?

Explain whether you are thinking about a career in science.

What is the name of the school you currently attend?

Figure S1. Handout used to assess student understanding of activity.
